# Diagnostic value of micrographia in Parkinson’s disease: a study with [^123^I]FP-CIT SPECT

**DOI:** 10.1007/s00702-022-02517-1

**Published:** 2022-05-27

**Authors:** Mikael Eklund, Simo Nuuttila, Juho Joutsa, Elina Jaakkola, Elina Mäkinen, Emma A. Honkanen, Kari Lindholm, Tero Vahlberg, Tommi Noponen, Toni Ihalainen, Kirsi Murtomäki, Tanja Nojonen, Reeta Levo, Tuomas Mertsalmi, Filip Scheperjans, Valtteri Kaasinen

**Affiliations:** 1 Clinical Neurosciences, University of Turku, FI-20014 Turku, Finland; 2 Neurocenter, Turku University Hospital, PO Box 52, FI-20521 Turku, Finland; 3 Turku PET Centre, Turku University Hospital, PO Box 52, FI-20521 Turku, Finland; 4 Turku Brain and Mind Center, University of Turku, FI-20014 Turku, Finland; 5 Clinical Medicine, Biostatistics, University of Turku and Turku University Hospital, PO Box 52, FI-20521 Turku, Finland; 6 Department of Clinical Physiology and Nuclear Medicine, Turku University Hospital, PO Box 52, FI-20521 Turku, Finland; 7 Department of Medical Physics, Turku University Hospital, PO Box 52, FI-20521 Turku, Finland; 8 HUS Medical Imaging Center, Clinical Physiology and Nuclear Medicine, University of Helsinki and Helsinki University Hospital HUS, PO Box 800, FI-00029 Helsinki, Finland; 9 Department of Neurology, Helsinki University Hospital HUS, Helsinki, Finland; 10 Department of Clinical Neurosciences, University of Helsinki HUS, PO Box 800, FI-00029 Helsinki, Finland

**Keywords:** Dopamine transporter, SPECT, Parkinson’s disease, Essential tremor, Micrographia

## Abstract

Micrographia is a common symptom of Parkinson’s disease (PD), and it may precede other motor symptoms. Despite the high prevalence of micrographia in PD, its neurobiological mechanisms are not known. Given that levodopa may alleviate consistent micrographia and that nondopaminergic essential tremor (ET) is not associated with micrographia, micrographia could possibly be used as an ancillary diagnostic method that reflects nigrostriatal dopamine function. We evaluated the usefulness of micrographia as a simple one-sentence writing test in differentiating PD from ET. A total of 146 PD patients, 42 ET patients and 38 healthy controls provided writing samples and were scanned with brain [^123^I]FP-CIT dopamine transporter (DAT) SPECT imaging with ROI-based and voxelwise analyses. The diagnostic accuracy of micrographia was evaluated and compared to that of DAT binding. Compared to ET and healthy controls, PD patients showed micrographia (consistent, 25.6% smaller area of handwriting sample in PD compared to ET, *p* = 0.002, and 27.2% smaller area of handwriting compared to healthy controls, *p* = 0.004). PD patients showed 133% more severe progressive micrographia compared with ET patients (median *b* =  − 0.14 in PD, *b* =  − 0.06 in ET, *p* = 0.021). In early unmedicated cognitively normal patients, consistent micrographia showed 71.2% specificity and 87.5% sensitivity in PD versus ET differentiation, but micrographia had no correlation with striatal or extrastriatal [^123^I]FP-CIT binding in patients with PD. The one-sentence micrographia test shows moderately good accuracy in PD versus ET differentiation. The severity of micrographia has no relationship with DAT binding, suggesting nondopaminergic mechanism of micrographia in PD.

ClinicalTrials.gov identifier: NCT02650843 (NMDAT study).

**Supplementary Information:**

The online version contains supplementary material available at 10.1007/s00702-022-02517-1.

## Introduction

Micrographia, defined as a decrease in the letter size of handwritten text (Larner 2011), was first described in association with neurological diseases by Pick (1903) and later connected to parkinsonism by Froment (1921). Micrographia is currently widely considered a core associated feature of Parkinson’s disease (PD) (Inzelberg et al. 2016), although estimates of its prevalence are highly variable (30–60%) (Wagle Shukla et al. 2012; Kim et al. 2005). Micrographia among other motor symptoms may precede phenoconversion to PD by up to 9–11 years (Fereshtehnejad et al. 2019), but the exact pathological mechanism remains unknown.

Micrographia can exist in a consistent or a progressive form. Consistent micrographia refers to a global decrease in letter size, whereas progressive micrographia refers to an initially normal but decreasing size while writing (Wilson 1925). However, it is evident that writing velocity, writing fluency and drawing (Kulkarni et al. 2013) may also deteriorate in PD, which suggests that PD is associated with general dysgraphia (Letanneux et al. 2014). Different pathogenetic mechanisms of consistent and progressive micrographia are supported by observations in levodopa-treated patients with PD: although levodopa seems to have efficacy in increasing handwriting size in patients with consistent micrographia (McLennan et al. 1972; Letanneux et al. 2014; Lange et al. 2006a, b), it appears to have no similar beneficial effect in patients with progressive micrographia (Ling et al. 2012). Findings with functional MRI have further supported different mechanisms of consistent and progressive micrographia in PD (Wu et al. 2016). Although previous studies support a dopaminergic mechanism underlying particularly consistent micrographia, there are no previous imaging studies on micrographia with dopaminergic tracers.

If micrographia is a specific early phenomenon of PD, it could play a role in the diagnostic process. The current diagnostic accuracy is suboptimal, as a marked proportion of patients with PD are misdiagnosed, particularly in the early stages of the disease (Adler et al. 2014; Joutsa et al. 2014). One of the difficulties in early diagnostics is the separation of tremor-dominant PD from nondopaminergic tremor syndromes, such as essential tremor (ET). Both the European Medicines Agency (EMA) and the US Food and Drug Administration (FDA) have approved striatal dopamine transporter (DAT) imaging as a tool for differentiating PD from ET (EMA 2007; FDA 2011), but DAT imaging involves radiation, is expensive and has limited availability. Given that micrographia may be connected to an early dysfunction of the dopamine system and that ET may be associated with graphia, the opposite of micrographia (Martinez-Hernandez et al. 2014), we hypothesized that the evaluation of micrographia could be used in PD versus ET diagnostics instead of, or together with, DAT imaging.

In this study, we therefore aimed to investigate the clinical diagnostic significance of micrographia in patients with PD compared to patients with ET, with a special focus on the diagnostic accuracy of testing for consistent and progressive micrographia. We also investigated possible connections between micrographia and brain DAT deficiency, a key neurobiological change in PD.

## Methods

### Participants

This prospective study included 146 patients with PD, 42 patients with ET and 38 healthy age- and sex-matched controls. Patients were chosen according to their diagnosis from consecutive series of 455 patients with clinically unclear Parkinsonian syndrome that were sent to a diagnostic brain [^123^I]FP-CIT SPECT imaging by a neurologist in Turku University Hospital or Helsinki University Medical Imaging Center, Finland (NMDAT study; ClinicalTrials.gov identifier: NCT02650843). Each participant provided an informed consent and was clinically examined 2–4 h prior to imaging. In addition to providing writing and drawing samples, examinations included a clinical interview, part III of the International Parkinson and Movement Disorder Society’s Unified Parkinson’s Disease Rating Scale (MDS-UPDRS) (Goetz et al. 2008), the Mini-Mental State Examination (MMSE) (Folstein et al. 1975) and the Beck Depression Inventory (BDI) (Beck et al. 1961). All examinators successfully completed the MDS-UPDRS Training Program and Exercise (MDS-UPDRS). Patients with MMSE scores less than 18 were excluded from the study. The demographic and clinical characteristics of the study participants are presented in Table 1. PD and ET patients were diagnosed by neurologists without knowledge of the results of writing sample measurements. Clinical diagnoses of PD and ET were confirmed after a mean (SD) clinical follow-up of 3.0 (1.6) years after imaging using full patient histories, including medication response, symptoms, signs, laboratory and imaging results. The MDS-UPDRS bradykinesia subscore includes finger and toe tapping, hand movements, pronation-supination movements of the hand, leg agility, raising from the chair, gait and global bradykinesia. Healthy controls were scanned using the same protocol, and each control participant underwent the same clinical examinations. The inclusion criteria for healthy controls were age 50–85 years, no medication affecting the central nervous system, and no neurological symptoms, or relevant prior neurological or psychiatric diseases. The study was accepted by the local ethics committee and was conducted according to the Declaration of Helsinki.

**Table 1:** Demographic and clinical characteristics of patients with PD and ET in comparison with healthy individuals (HC)

Variable group	Variable	PD	ET	HC	*P* value^a^
Demographics	*n*	146	42	38	–
	Age, years	66.0 (15)	66.0 (17.0)	68.0 (14.0)	0.420
	Sex, male/female	73/73	20/22	19/19	0.962
	Handedness, right/left/symmetrical	135/7/4	36/3/3	35/2/1	0.663
	Levodopa-treated, yes/no	28/118	1/41	0/38	< 0.001
	LEDD, mg	381 (158)^b,c^	200 (0)	–	< 0.001
Motor symptoms	MDS-UPDRS III motor score	34.0 (21.5)^c^	33.5 (20.3)^c^	5.5 (7.3)	< 0.001
	MDS UPDRS III bradykinesia subscore	18.5 (13.0)^c^	15.0 (11.0)^c^	4.0 (6.0)	< 0.001
	Predominant side of motor symptoms, right/left/symmetrical	24/36/86	5/1/36	–	0.002
	Motor symptom duration, months	18.0 (25.0)	27.0 (96.0)	–	< 0.001
Cognition	MMSE	28.0 (3.0)	28.0 (3.0)	28.0 (2.0)	0.160
Depression	BDI	6.0 (7.0)	6.8 (9.1)	1.0 (6.0)	< 0.001
Micrographia	Consistent, mean height of letters, mm	4.3 (1.9)^b,c^	5.0 (1.7)	5.0 (1.4)	< 0.001
	Consistent, mean area of writing sample, mm^2^	399 (301)^b,c^	536 (397)	548 (286)	< 0.001
	Progressive, *b* value	− 0.14 (0.26)^b^	− 0.06 (0.18)	− 0.06 (0.32)	0.014
	Drawing, cm^2^	22.8 (25.4)	26.7 (22.6)	25.8 (28.0)	0.485
DAT SBR	Caudate nucleus	2.08 (0.78)^b,c^	3.09 (1.23)^c^	2.58 (0.54)	< 0.001
	Anterior putamen	1.56 (0.78)^b,c^	2.96 (1.07)	2.50 (0.60)	< 0.001
	Posterior putamen	0.90 (0.56)^b,c^	2.53 (0.87)	2.18 (0.59)	< 0.001
	Right posterior putamen	0.93 (0.61)^b,c^	2.60 (1.00)	2.22 (0.55)	< 0.001
	Left posterior putamen	0.89 (0.54) ^b,c^	2.58 (0.90)	2.17 (0.51)	< 0.001

Values are medians (IQR). One patient with ET was treated with levodopa at the time of imaging, before the diagnosis was confirmed

^a^Kruskal-Wallis test, Pearson Chi-square test or Fisher’s exact test as appropriate

^b,^^c^^,d^Significantly different compared to ET (b) or HC (c) in pairwise comparison with Kruskal–Wallis test adjusted by the Dunn-Bonferroni’s correction for multiple test

*PD* Parkinson’s disease, *ET* essential tremor, *HC* Healthy control, *MMSE* Mini Mental State Examination, *BDI* Beck’s Depression Inventory

### Evaluation of micrographia

**Fig. 1:**
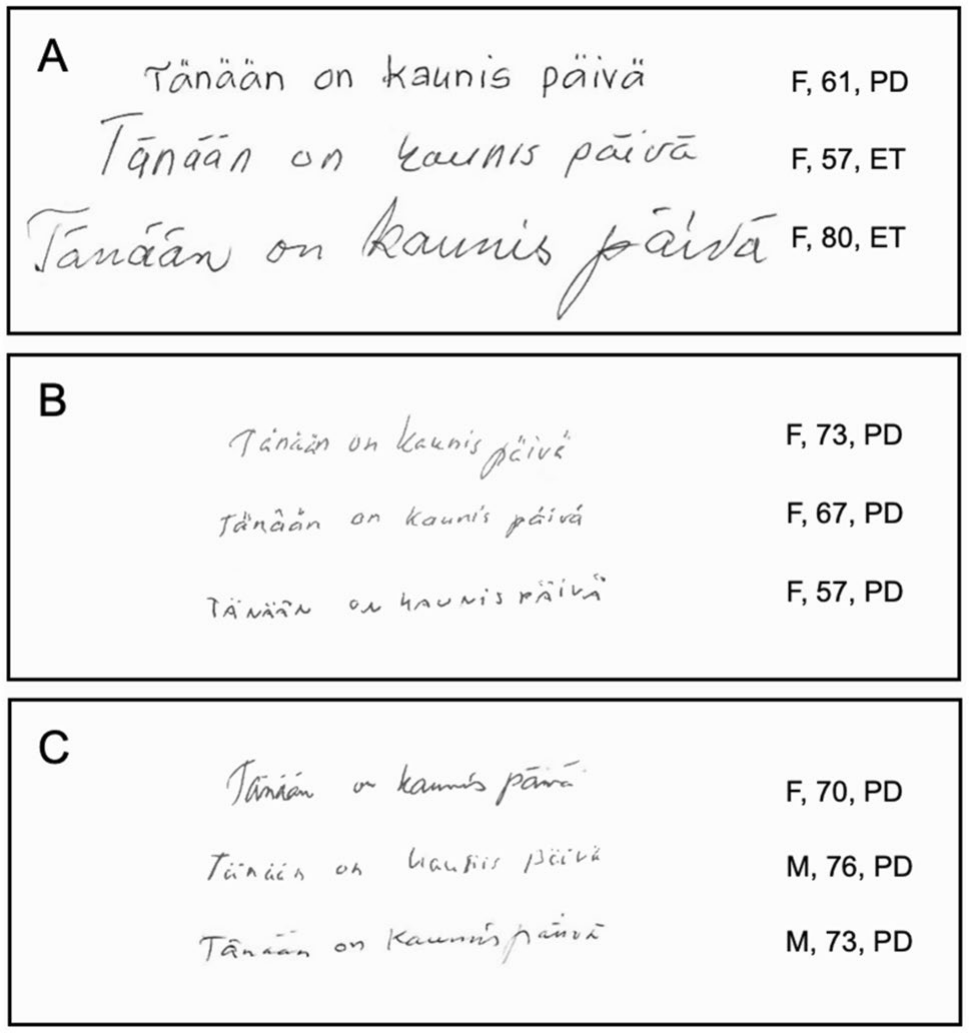
Representative examples of consistent and progressive micrographia in studied participants. **A** No micrographia **B** Consistent micrographia (global decrease in letter size). **C** Progressive micrographia (initially normal but decreasing size). Demographic information included sex (M/F), age (years) and diagnosis (PD/ET)

Writing and drawing tasks were performed in a standardized manner for each subject in the two centers prior to scanning (same time of day, room, lighting, chair and desk). Samples were written and drawn on a blank white paper using a pen and the subjects were instructed to draw a house with windows and a door, with a sentence “Tänään on kaunis päivä” which translates to English as “Today is a beautiful day” under the drawn house (Fig. 1). The sentence was selected for its simplicity, briefness and clinical applicability. The height of the letters “T”, “p” and “ä”, without the umlaut, were measured in addition to the width of the writing sample using a standard ruler. In total 57 subjects from 455 scanned subjects were excluded from the study due to missing or incomplete writing or drawing sample or both. The mean height of the letters and area of the writing sample (mean height of the letters multiplied by the width of the writing sample) were calculated as a measure of consistent micrographia. To evaluate progressive micrographia in the writing samples, the heights of the letters “a” were plotted from the first to the sixth, and the regression line of “a” letter heights was obtained for each participant. The slope of the regression line (*b* value) was used as a measurement for progressive micrographia (Kim et al. 2005; Ling et al. 2012; Wu et al. 2016). The width and height of the house were measured to calculate the area of the drawing sample. Incomplete writing samples or samples written in two rows were excluded. We used previously described criteria for micrographia defined as below − 2 SD mean letter height for consistent micrographia and − 2 SD mean regression line *b* value for progressive micrographia compared to healthy controls (Wu et al. 2016). The reproducibility of measurements was verified with intraclass correlation coefficients (ICCs) using data from 54 participants (including patients with PD and ET, and healthy controls), as measured by two examiners (M.E. and K.L.) (Koo et al. 2016).

### Imaging and image analysis

An injection of 185 MBq of [^123^I]FP-CIT was given to participants three hours prior to SPECT imaging. To prevent exposure of the thyroid tissue to radiation, potassium perchlorate (250–300 mg) or Jodix™ tablets (130 mg) were given 30–60 min before the injection. SPECT imaging was performed using one of our eight SPECT/CT devices. All SPECT/CT devices were calibrated using a striatal phantom (RSD, Radiology Support Devices, Inc., Long Beach, USA) before the study to allow data comparison. The calibration procedure followed the guidance of Hermes Medical Solutions (Diemling et al. 2012; Tossici-Bolt et al. 2011). The SPECT data were reconstructed using the 3D OSEM algorithm with attenuation, collimator response and scatter corrections (HybridRecon Neurology, version 1.3, Hermes Medical Solutions AB, Stockholm, Sweden). The acquisition and reconstruction protocols were based on EANM recommendations (Darcourt et al. 2010) for all our devices. Reconstructed images were analyzed using BRASS semiautomated analysis software (version 2.6, Hermes Medical Solutions, Stockholm, Sweden), and scanner-specific corrections based on our calibrations were used for the BRASS analyses (Diemling et al. 2012; Tossici-Bolt et al. 2011; Varrone et al. 2013). Specific binding ratios (SBRs) for six regions of interest (left and right caudate, anterior putamen and posterior putamen) were calculated using the occipital cortex as the reference tissue: SBR = (VOI _caudate or putamen_ – VOI _occipital_)/VOI _occipital_ (Darcourt et al. 2010).

Voxelwise analyses were conducted to investigate the association between measures of micrographia and striatal and extrastriatal [^123^I]FP-CIT binding in patients with PD. Image preprocessing and voxelwise statistical analyses were conducted using Statistical Parametric Mapping software (SPM12; SPM12 2022). A nonlinear transformation was computed from the average of all reconstructed scans to an in-house [^123^I]FP-CIT SPECT template (Kaasinen et al. 2015). This transformation was applied to all individual reconstructed images, and the normalization results were inspected visually. As in BRASS analyses, the occipital cortex was used as the reference tissue to calculate region-to-occipital cortex uptake ratio images, which were then smoothened using an 8-mm Gaussian kernel. A general linear model with age and sex, with and without UPDRS motor score as covariates, was created to investigate the association between DAT binding and micrographia severity (consistent or progressive). An analysis mask, including the frontal lobes, cingulate cortex, basal ganglia, thalamus, medial temporal lobe and midbrain, was created using WFU pickatlas (version 3.05) to exclude brain regions with negligible [^123^I]FP-CIT binding from the analyses. Cluster-level familywise error (FWE)-corrected *p* values at a height threshold of *p* < 0.001 were considered significant (WFU_PickAtlas 2022).

### Statistical analyses

IBM SPSS Statistics (version 26, SPSS, Inc., Chicago, Illinois, USA) was used for all statistical analyses except for voxelwise analyses. Normal distribution of the variables was confirmed using the Shapiro–Wilk test, and the intraclass correlation coefficient (ICC) was used for the reliability analysis (Koo et al. 2016). A two-way random-effect model based on single ratings and absolute agreement assessed the interrater repeatability. Mean estimations for single measures along with 95% confidence intervals (CIs) were obtained for each measurement. ICC values were interpreted as follows: below 0.50 as poor; 0.50–0.75 as moderate; 0.75–0.90 as good; and above 0.90 as excellent reliability (Koo et al. 2016).

For comparisons among three groups, chi-square, Fisher’s exact, and Kruskal–Wallis tests were used, followed by pairwise testing with Dunn-Bonferroni’s corrections as appropriate. To investigate relationships between individual parkinsonian motor symptoms and DAT binding laterality, patients were categorized according to lateralized symptoms as described (Kaasinen 2016). Analysis of covariance (ANCOVA), adjusted for age, sex, cognition, and motor symptom severity, was used to compare patients with PD with and without micrographia. Spearman’s rho correlation coefficients were used to assess relationships between SBRs, micrographia, age, cognition, depression, and motor symptom severity. Receiver operating characteristic (ROC) curve analysis and Youden index were used to examine the diagnostic sensitivity and specificity of micrographia (Hajian-Tilaki 2013).

## Results

### Group differences in micrographia and DAT binding

**Fig. 2:**
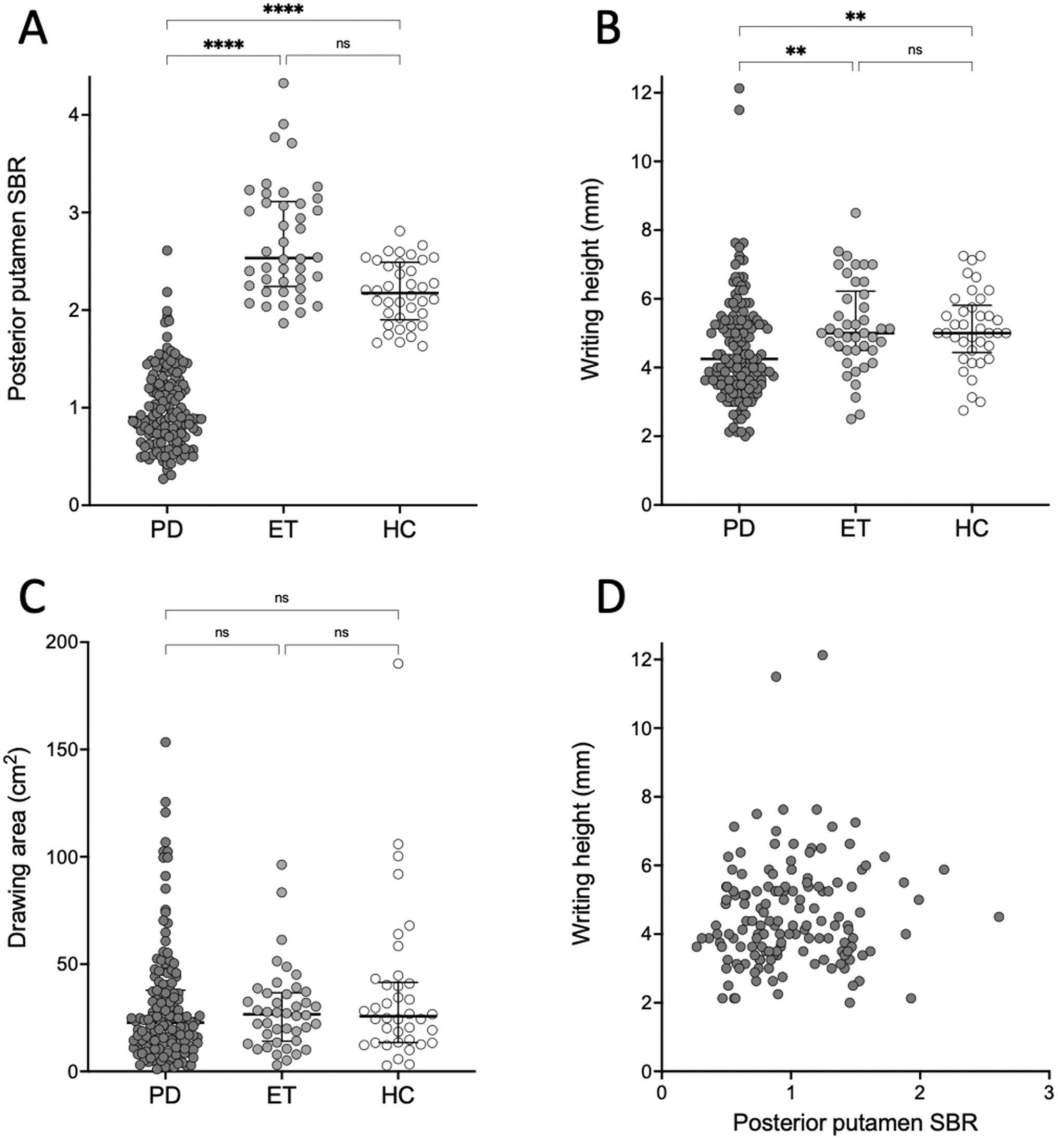
Micrographia measurements and specific binding ratios of DAT binding. **A** Mean posterior putamen specific binding ratio (SBR) of DAT binding in PD patients (*n* = 146), ET patients (*n* = 42) and healthy controls (HCs, *n* = 38). **B** Mean height of measured letters. **C** Area of drawing samples. **D** There was no correlation between the mean posterior putamen SBR and the mean height of the measured letters in PD patients (Spearman *r* = 0.12, *p* = 0.16). Medians and IQR are marked with horizontal lines. ****p < 0.0001, **p < 0.01 and *ns* not significant

Patients with PD wrote 14.0% smaller letters, and the area of the writing sample was 25.6% smaller than that of patients with ET (Fig. 1, Fig. 2, Table 1; Kruskal–Wallis *p* < 0.001; in pairwise comparison PD vs. ET Dunn-Bonferroni-corrected *p* < 0.006). Compared to healthy controls, the corresponding relative differences were 14.0% and 27.2%, respectively (PD vs. healthy controls, corrected *p* < 0.008). There were no differences in letter height or area of writing sample between healthy controls and patients with ET (*p* = 1.0). Patients with PD had 133% more severe progressive micrographia than did patients with ET (*b* =  − 0.14 in PD, *b* =  − 0.06 in ET, corrected *p* = 0.021), without a difference between patients with PD and healthy controls (Table 1; *p* = 0.28). There were no differences in the slope of the regression line between ET and healthy controls (Table 1; *p* = 1.0) nor in drawing micrographia (PD vs. ET vs. healthy controls; Table 1; *p* = 0.49). The analysis was separately carried out in patients with MMSE scores =  > 24 and the results remained essentially the same (Supplementary Table 1). From 146 PD patients, 28 were medicated with levodopa. There were no differences in any micrographia measurement between levodopa-medicated and unmedicated PD patients (*p* > 0.24). There were no differences in any micrographia measurement between ET patients with rest tremor (UPDRS rest tremor higher than or equal to 2) or without rest tremor (UPDRS rest tremor score less than 2) (*p* > 0.076).

In DAT binding, patients with PD had markedly lower tracer uptake in all studied striatal brain regions than both patients with ET and healthy controls (corrected *p* < 0.001, Fig. 2). Posterior putamen DAT binding was 64.4% lower in patients with PD than in patients with ET and 58.7% lower in patients with PD than in healthy controls. Laterality in motor symptoms or in DAT binding was not associated with consistent or progressive micrographia (right-handed participants; *p* > 0.49).

### Correlations between micrographia, DAT binding and clinical characteristics

Micrographia (consistent, progressive or drawing) did not correlate with striatal DAT binding in any group, in any of the studied brain regions (0.21 > *r* >  − 0.24, *p* > 0.18) (Fig. 2). In patients with PD, consistent micrographia as the mean height of letters correlated with the MDS-UPDRS motor score (*r* =  − 0.21, *p* = 0.01) and bradykinesia score (*r* =  − 0.21, *p* = 0.012) (decrease in letter size associated with higher severity score). In patients with PD, striatal DAT binding correlated with the MDS-UPDRS motor score (− 0.28 > *r* >  − 0.43, *p* < 0.001) and bradykinesia (0.44 > *r* >  − 0.30, *p* < 0.001) in all studied brain regions. There were no correlations between mean height of letters or area of the handwriting sample and striatal DAT binding in any of the studied brain regions in PD patients with consistent micrographia (*n* = 13, Spearman − 0.253 < *r* < 0.152, *p* > 0.404) or between b-value of the regression line and striatal DAT binding in any of the studied brain regions in PD patients with progressive micrographia (*n* = 13, Spearman − 0.296 < *r* < 0.011, *p* > 0.326) (Supplementary Fig. 1).

### Voxelwise analyses

There were no significant associations between tracer binding and consistent or progressive micrographia in patients with PD in striatal or extrastriatal regions, as studied with SPM.

### Group differences between patients with and without micrographia

Thirteen patients with PD had consistent micrographia, and thirteen patients with PD had progressive micrographia using the – 2 SD criteria. There were no patients expressing both forms of micrographia. There were no differences in DAT binding, age, cognition, or depression between patients with PD with or without consistent or progressive micrographia. Patients with PD with consistent micrographia had more severe motor symptoms (*p* = 0.017) and more severe bradykinesia (*p* = 0.015) than patients with PD without consistent micrographia. In contrast, patients with PD with progressive micrographia had fewer motor symptoms than patients without progressive micrographia (*p* = 0.012), without a difference in bradykinesia (*p* = 0.103). Two patients with ET had consistent micrographia, and two separate patients with ET had progressive micrographia. These patients had more motor symptoms than other ET patients (UPDRS motor score mean (SD): 48.5 (9.5)) suggesting that the phenotype of these patients was ET plus.

### Sensitivity and specificity analyses

Across the whole sample of patients with PD and ET, consistent micrographia in terms of the area of the writing sample showed 50.0% sensitivity and 78.6% specificity (ROC AUC 0.673, CI 95% 0.584–0.762, cutoff point 397.9 mm^2^), and the mean height of letters showed 56.8% sensitivity and 78.6% specificity (ROC AUC 0.657, CI 95% 0.568–0.745, cutoff point 4.44 mm) for PD vs. ET. Progressive micrographia showed 52.1% sensitivity and 78.6% specificity (ROC AUC 0.651, CI 95% 0.554–0.727, cutoff point − 0.13). In unmedicated patients with motor symptom durations less than or equal to 24 months, MMSE scores greater than or equal to 27 and MDS-UPDRS scores less than or equal to 36 (representing early disease; PD 39 patients and ET 8 patients), consistent micrographia had 92.3% sensitivity and 62.5% specificity (ROC AUC 0.824, CI 95% 0.690–0.957, cutoff point 5.94 mm), while the area of the writing sample showed 71.2% sensitivity and 87.5% specificity (ROC AUC = 0.862, CI 95% 0.734–0.990, cutoff point 525 mm^2^) for PD versus ET.

### Validation

The interrater ICC (single measures) of micrographia measurements ranged between 0.845 and 0.977, with a 95% confidence interval for the lower boundary ranging between 0.602–0.925 and the upper boundary ranging between 0.925–0.979 for all micrographia measurements.

## Discussion

Our study focused on PD and ET, two movement disorders that may be difficult to differentiate in the early stages. There are three primary results of the study. First, the results show that, as a group, patients with PD have more both consistent and progressive micrographia in free writing compared with patients with ET. Second, in early stages, the PD versus ET diagnostic accuracy of micrographia is moderately good, but the accuracy deteriorates as the symptoms progress. Third, striatal DAT binding deficiency does not have any correlation with micrographia, pointing to a primarily nondopaminergic mechanism of micrographia in PD.

Patients with PD in our study had clearly smaller handwriting compared to patients with ET, but there was no difference between patients with ET and healthy controls. The similar writing size in patients with ET and healthy individuals thus demonstrates that the difference between PD and ET is not due to the suggested macrographia in ET (Martinez-Hernandez et al. 2014), but that the effect is driven by PD pathology. Furthermore, based on our results, the defect in PD seems to be specifically associated with writing and not with drawing. The process of handwriting with learned oscillatory-like small-amplitude movements thus appears particularly vulnerable in PD compared with the larger directional hand movements involved in drawing. The accuracy of testing for micrographia was moderately good in detecting PD versus ET when patients with early stages of the disease were included in the analysis (71.2% sensitivity and 87.5% specificity), indicating that, in clinical practice, micrographia has diagnostic value only in early-stage tremor patients (symptom duration <  = 24 months, unmedicated, UPDRS motor score <  = 36, MMSE =  > 27). Nevertheless, diagnostic separation using DAT imaging is superior in sensitivity (98% sensitivity and 68% specificity in early PD) (de la Fuente- Fernández 2012) compared to micrographia measurements, demonstrating that writing evaluations should not replace dopaminergic imaging, although it represents a simple, easily available method to gain additional diagnostic information at the bedside. The presented diagnostic cutoffs can be considered language specific for Finnish, and corresponding values for other languages would need to be estimated. It is also possible that a letter copying task, which also seems to detect micrographia well (Inzelberg 2016), involving drawing shapes repetitively or a longer writing sample, could provide even higher value in diagnostic separation. Effects of tremor on writing or drawing size were not specifically investigated. It is also worth noting that the cut-off values used with our sample, based on previous literature, may not be the most accurate in identifying individuals with relevant micrographia. However, we compared the results using several different cut-off values and the results remained essentially the same (data not shown).

It is notable, that MDS-UPDRS motor scores were high in ET patients of our study pointing to a possibility that many ET patients of our study were ET plus phenotype (Bhatia et al. 2018) rather than being purely tremulous ET. This may be caused by selection bias of physicians sending patients to diagnostic SPECT scan, since SPECT scan may not have been required for diagnostic of purely tremulous ET with no other motor symptoms. Phenotypic overlap of ET plus and PD is underlined in our study, since 4 ET patients having consistent or progressive micrographia also had higher UPDRS motor scores than other ET patients in our study.

Together with the diagnostic performance of micrographia per se, we investigated possible connections with basal ganglia dopamine function in PD. It is notable that micrographia may to occur also in other neurodegenerative diseases such as Huntington’s disease or corticobasal degeneration, or lesions of basal ganglia (Inzelberg et al. 2016), that do not necessarily present with parkinsonism. No imaging studies with dopaminergic tracers have been conducted on micrographia before. In previous studies, consistent micrographia has shown some improvement after the initiation of levodopa therapy (McLennan et al. 1972), decrement of handwriting kinematics shown after withdrawal of patients usual dopaminergic treatment (Lange, Tucha et al. 2006a, b) and focal basal ganglia lesions have been reported to be associated with micrographia (Inzelberg et al. 2016). We therefore suspected that micrographia would show at least modest correlations with striatal dopamine function. Instead, we did not observe any relationships between striatal DAT binding or extrastriatal tracer binding and writing. This points to primarily nondopaminergic mechanisms in micrographia development in PD. For instance, if micrographia was merely a reflection of upper extremity rigidity in PD and rigidity is associated with striatal DAT binding (Pirker 2003), then this association would probably have been detected with our large sample of patients with PD who provided writing samples and were examined with MDS-UPDRS and DAT SPECT. Even with the large sample size and considerable variability in micrographia, plus DAT binding and symptom severity measures in the three groups, there was no link between micrographia and presynaptic striatal dopaminergic function. Nevertheless, our results support the theory that the mechanisms of consistent and progressive micrographia in PD differ, as patients with consistent micrographia showed more severe motor symptoms and bradykinesia than did patients without consistent micrographia, whereas the effect was the opposite for progressive micrographia. Indeed, a recent functional MRI study has suggested that consistent micrographia is connected to decreased activity and connection in the basal ganglia motor circuit, whereas progressive micrographia is connected to disconnections between the rostral supplementary motor area, rostral cingulate motor area and cerebellum, in addition to dysfunction of the basal ganglia motor circuit (Wu et al. 2016). Although our results are in line with the concept of different mechanisms of consistent and progressive micrographia in PD, the current conclusions are inconclusive with respect to symptom severity and different forms of micrographia (Kim et al. 2005). The frequency of consistent and progressive micrographia in our patients with PD was also higher (9%) than that in a previous study (3%) that used the same criteria for micrographia (Kim et al. 2005). The difference in the prevalence is probably due to differences in the selection of patients. Our sample was collected from mostly unmedicated, including some levodopa-treated, de novo patients who were referred for DAT imaging due to diagnostic uncertainty, whereas patients in the earlier study were medicated patients with PD with a mean disease duration of 5.2 years. It is possible that the use of dopaminergic medication alleviated consistent micrographia to normal levels in the previous study, as has been reported (McLennan et al. 1972; Letanneux et al. 2014; Lange et al. 2006a, b). Another possibility is that, in contrast to the Latin alphabet, the previous study used Korean characters to assess micrographia, which could have affected the prevalence if the required writing movements are less oscillatory. It is possible that the prevalence of clinically relevant micrographia might have higher, had a longer, repetitive writing task of single letters or longer sentences been used. In addition, it is possible that electronic writing tablets, instead of paper and pen, can capture important additional writing kinematics, such as writing velocity and fluency, which may be particularly useful in PD diagnostics.

To conclude, our results demonstrate that in early-stage tremor patients, a simple micrographia measurement can assist in the differentiation between PD and ET. DAT imaging remains superior in sensitivity (Hajian-Tilaki 2013), but micrographia can have value as an ancillary diagnostic test. The results also indicate that writing dysfunction in PD is not related to presynaptic dopaminergic function, at least when measured with DAT imaging. Further studies are needed to investigate the connection between writing kinematics and other neurobiological mechanisms in PD, as well as the diagnostic value of micrographia in other neurodegenerative diseases.

## Supplementary Information

Below is the link to the electronic supplementary material.

Supplementary file1 (PDF 401 kb)

## Data Availability

Anonymized data not published within this article will be made available by request from any qualified investigator.
