# Supplementary material for: Diagnostic value of micrographia in Parkinson’s disease: a study with [123I]FP-CIT SPECT
Source: J Neural Transm (Vienna). 2022 May 27;129(7):895–904. doi: 10.1007/s00702-022-02517-1 (PMC9217822; doi:10.1007/s00702-022-02517-1)
Supplement: Supplementary file 1 — Supplementary file1 (PDF 401 kb) [file 702_2022_2517_MOESM1_ESM.pdf]

Diagnostic value of micrographia in Parkinson's disease: A study with [^{123}I]FP-CIT SPECT

Mikael Eklund BM[1,2,3], Simo Nuuttila BM[1,2], Juho Joutsa MD, PhD[1,2,3,4], Elina Jaakkola MD, PhD[1,2], Elina Mäkinen MD, PhD[1,9], Emma A. Honkanen MD[1,2,3], Kari Lindholm RN[1,2], Tero Vahlberg MSc[5], Tommi Noponen PhD[6,7], Toni Ihalainen PhD[8], Kirsi Murtomäki MD[9], Tanja Nojonen RN[9], Reeta Levo RN[9], Tuomas Mertsalmi MD[9], Filip Scheperjans MD, PhD[9], Valtteri Kaasinen MD, PhD[1,2,4]

*Corresponding author

1. Clinical Neurosciences, University of Turku, FI-20014 University of Turku, Turku, Finland
2. Neurocenter, Turku University Hospital, PO Box 52, FI-20521 Turku, Finland
3. Turku PET Centre, Turku University Hospital, PO Box 52, FI-20521 Turku, Finland
4. Turku Brain and Mind Center, University of Turku, FI-20014 University of Turku, Turku, Finland
5. Clinical Medicine, Biostatistics, University of Turku and Turku University Hospital, PO Box 52, FI-20521 Turku, Finland.
6. Department of Clinical Physiology and Nuclear Medicine, Turku University Hospital, PO Box 52, FI-20521 Turku, Finland
7. Department of Medical Physics, Turku University Hospital, PO Box 52, FI-20521 Turku, Finland
8. HUS Medical Imaging Center, Clinical Physiology and Nuclear Medicine, University of Helsinki and Helsinki University Hospital, PO Box 800, FI-00029 HUS, Finland
9. Department of Neurology, Helsinki University Hospital and Department of Clinical Neurosciences, University of Helsinki, PO Box 800, FI-00029 HUS, Finland

Corresponding author: Mikael Eklund (ORCID iD: 0000-0002-1947-712X)

Address: Clinical Neurosciences, University of Turku, FIN-20014 University of Turku, Turku, Finland

Telephone: +358 2 313 2700

Email: mreekl@utu.fi

Supplementary material

- **Supplementary Fig. 1**
- **Supplementary Fig. 2**
- **Supplementary Table 1**

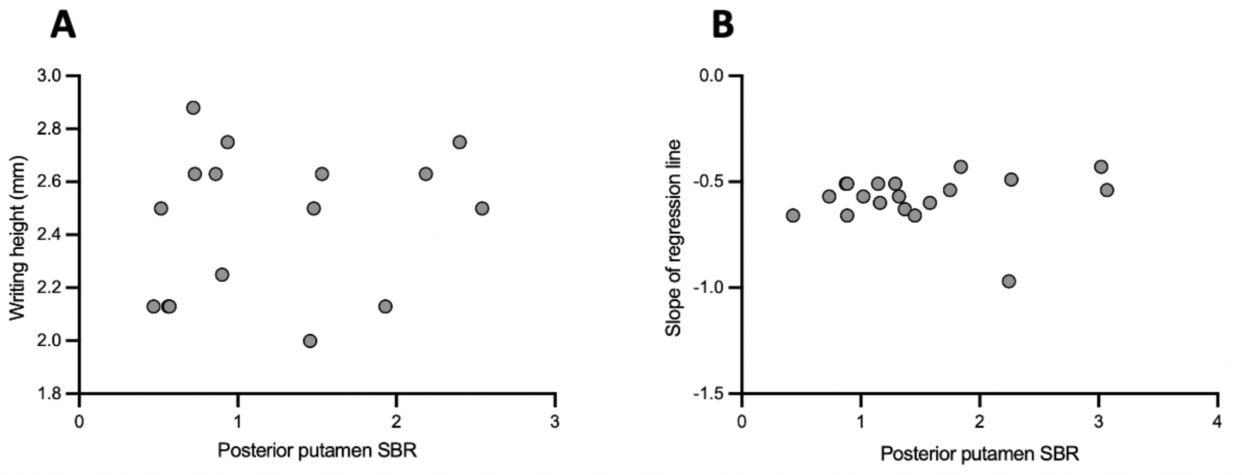

Supplementary Fig. 1. Correlations between micrographia measurements and specific binding ratios of DAT binding in PD patients with micrographia. A. There was no correlation between the mean posterior putamen SBR and the mean height of the measured letters in PD patients with consistent micrographia (n=13; Spearman r=0.084, p=0.79). B. There was no correlation between the mean posterior putamen SBR and the slope of the regression line in PD patients with progressive micrographia (n=13; Spearman r=-0.16, p=0.60).

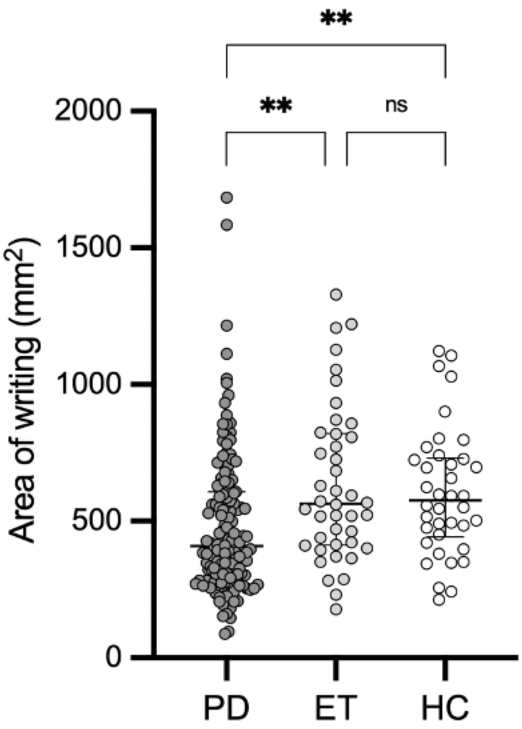

Supplementary Fig. 2. Area of writing sample (mm2) and specific binding ratios of DAT binding in PD patients (n=146), ET patients (n=42) and healthy controls (HCs, n=38). Medians and IQR are marked with horizontal lines. **p<0.01 and ns=not significant

Supplementary Table 1. Group differences in writing and drawing tests in patients with MMSE scores => 24 Values are median (IQR).

	**PD (n=134)**	**ET (n=41)**	**HC (n=36)**	**P-value[a]**
Consistent, mean height of letters, mm	4.2 (1.8)[b,c]	5.0 (1.8)	5.0 (1.6)	<0.001
Consistent, mean area of writing sample, mm^2	392 (302)[b,c]	536 (402)	560 (306)	<0.001
Progressive, b-value	-0.14 (0.26)[b]	-0.06 (0.14)	-0.03 (0.32)	0.014
Drawing, cm^2	23.1 (26.6)	27.1 (21.0)	26.8 (27.8)	0.323

a = Kruskall-Wallis test
b = significant Bonferroni corrected p-value vs ET
c = significant Bonferroni corrected p-value vs HC
